# Asymmetric Morphological Priming Among Inflected and Derived Verbs and Nouns in Greek

**DOI:** 10.3389/fpsyg.2021.658189

**Published:** 2021-11-17

**Authors:** Sofia Loui, Athanassios Protopapas, Eleni Orfanidou

**Affiliations:** ^1^Department of History and Philosophy of Science, National and Kapodistrian University of Athens, Athens, Greece; ^2^Department of Special Needs Education, University of Oslo, Oslo, Norway; ^3^Department of Psychology, American College of Greece, Athens, Greece

**Keywords:** lexical representation, lexical decision, modern Greek language, inflectional morphology, derivational morphology, grammatical class, masked priming, long-lag priming

## Abstract

The present study examined differences between inflectional and derivational morphology using Greek nouns and verbs with masked priming (with both short and long stimulus onset asynchrony) and long-lag priming. A lexical decision task to inflected noun and verb targets was used to test whether their processing is differentially facilitated by prior presentation of their stem in words of the same grammatical class (inflectional morphology) or of a different grammatical class (derivational morphology). Differences in semantics, syntactic information, and morphological complexity between inflected and derived word pairs (both nouns and verbs) were minimized by unusually tight control of stimuli as permitted by Greek morphology. Results showed that morphological relations affected processing of morphologically complex Greek words (nouns and verbs) across prime durations (50–250ms) as well as when items intervened between primes and targets. In two of the four experiments (Experiments 1 and 3), inflectionally related primes produced significantly greater effects than derivationally related primes suggesting differences in processing inflectional versus derivational morphological relations, which may disappear when processing is less dependent on semantic effects (Experiment 4). Priming effects differed for verb vs. noun targets with long SOA priming (Experiment 3), consistent with processing differences between complex words of different grammatical class (nouns and verbs) when semantic effects are maximized. Taken together, results demonstrate that inflectional and derivational relations differentially affect processing complex words of different grammatical class (nouns and verbs). This finding indicates that distinctions of morphological relation (inflectional vs. derivational) are not of the same kind as distinctions of grammatical class (nouns vs. verbs). Asymmetric differences among inflected and derived verbs and nouns seem to depend on semantic effects and/or processing demands modulating priming effects very early in lexical processing of morphologically complex written words, consistent with models of lexical processing positing early access to morphological structure and early influence of semantics.

## Introduction

A considerable body of research on the nature of lexical representation and processing suggests that words are represented and stored in memory in terms of their morphological constituents (play-er, play-ing, dis-play). These constituents are used in processes of language production (Levelt et al., 1999) and comprehension (e.g., Feldman and Andjelkovic, 1992; Marslen-Wilson et al., 1994; Sandra and Taft, 1994), such that all complex words are composed of their constituent morphemes during word production and decomposed into them during comprehension.

Evidence for this morpheme-based representation view comes from studies, suggesting that morphemic structure influences lexical processing of written words: lexical processing times are facilitated by frequency of root (Colé et al., 1989; Baayen et al., 1997; Alegre and Gordon, 1999), by frequency of suffix (though marginally; English: Juhasz et al., 2003; Italian: Burani D. et al., 1997; Burani A. et al., 2006; Burani and Thornton, 2003), and by prior presentation of a morphologically related word (e.g., player-PLAY; Forster et al., 1987; Grainger et al., 1991; Marslen-Wilson et al., 1994; Frost et al., 1997; Rastle et al., 2000; Crepaldi et al., 2010). These facilitatory effects are argued to support a distinct role of morphemic structure in lexical processing that is independent of systematic relations between form and meaning (for a different view see Gonnerman et al., 2007; Baayen et al., 2011; Milin et al., 2017).

In line with this view, morphological processing models posit obligatory decomposition of complex words, either prior to lexical access (Rastle et al., 2004; Taft and Nguyen-Hoan, 2010) or following lexical access of whole words (Giraudo and Grainger, 2001, 2003; Järvikivi et al., 2009).

However, it remains controversial whether all morphologically complex words are represented in a decomposed manner. Complex words consisting of roots and derivational suffixes (e.g., player) are proposed to be accessed and represented as whole words, as opposed to those consisting of roots and inflectional suffixes (e.g., play-s) (Stanners et al., 1979; Friederici et al., 1989; Laudanna et al., 1992; Feldman, 1994; Marslen-Wilson et al., 1994; Marslen-Wilson, 2007; Klimovich-Gray et al., 2017; for a different view: Fowler et al., 1985; Bertram et al., 2000; Raveh and Rueckl, 2000; for a comprehensive review of relevant neuroimaging research: Leminen et al., 2019).^1^ Processing differences between derived and inflected words are consistent with models of lexical processing positing both word-based (for derived word forms; especially for semantically transparent forms with productive affixes) and morpheme-based (for inflected word forms) lexical representations (Caramazza et al., 1988; Marslen-Wilson et al., 1994; Schreuder and Baayen, 1995; Baayen et al., 1997). Alternatively, base morphemes of inflected forms (e.g., play in play-s) may be represented and processed differently from derived forms (e.g., play in player) because different levels of morphological representation are processed at different times during lexical processing: inflectional suffixes are believed to be processed before derivational ones (Laudanna et al., 1992).

These proposed differences in representation between inflectional and derivational forms may not reflect a simple categorical distinction between inflection and derivation processes. Rather, they may reflect mainly syntactic/grammatical and semantic processing differences. Specifically, derived word forms exhibit less systematic relationships between form and meaning than regularly inflected word forms. For example, the meaning of the derivational form -er in words such as “player” (cf. -er in corner) is typically less transparent than the inflectional form -s in “plays.” Derivation involves a change in meaning (e.g., govern-government vs. govern-governs), and it can alter grammatical class membership while inflection never does (e.g., play-player vs. play-plays). In addition, derivational suffixes: (a) are far less productive (and varying in productivity, e.g., -er in player vs. -th in warmth) than inflectional suffixes (e.g., -s for plural), (b) they do not mark syntactic features as inflectional suffixes do (e.g., number for nouns and tense for verbs), (c) they impact thematic role assignment (e.g., drive-driver), and (d) they do not have syntactic functions, in contrast to inflectional suffixes, which facilitate agreement (e.g., The girl eat-s vs. the girl-s eat). Finally, (e) derived word forms (drive→ driver) may also participate in inflection (driver-s), whereas the opposite is not true.

There is considerable evidence in linguistics, psycholinguistics, neuropsychology, and neuroimaging (Damasio and Tranel, 1993; Hillis and Caramazza, 1995; Sereno, 1999; Davis et al., 2004; Aggujaro et al., 2006; Pinker, 2009), suggesting that words belonging to different grammatical classes (especially nouns and verbs) are functionally distinct elements and therefore that grammatical class constitutes an organizational principle of lexical knowledge. However, alternative accounts in all of these fields (Sapir, 1921; Bates and MacWhinney, 1982; Cotelli et al., 2006; Longe et al., 2007; Crepaldi et al., 2014) reject this representational distinction between nouns and verbs as grammatical classes and claim that the observed differences arise as a consequence of different semantic and syntactic constraints. Specifically, verbs typically impose greater semantic processing demands than nouns, as verbs refer to events with internal structure involving additional, potentially multiple, entities, whereas nouns typically refer to objects or individual discrete entities. Verbs also impose greater syntactic processing demands than nouns, as they typically assign thematic roles in the sentence (e.g., the thematic role of an agent), whereas nouns can only receive a thematic role, but not assign one. Alternatively, the distinction between verbs and nouns may be ascribed to differences in morphological complexity of the grammatical information they incorporate: verbs are morphologically more complex than nouns, e.g., verbs have more inflected forms than nouns, and this can vary across languages.

The study of morphological structure and processing suggests that the distinction between the representation of inflected and derived words may be closely related to the distinction between the representation of different classes of words (nouns and verbs) and their grammatical properties. The relation concerns the role of grammatical class in lexical organization insofar as differences both between inflection and derivation processes and between nouns and verbs may reflect either categorical distinctions or semantic, syntactic, or purely morphological processes. The present study simultaneously takes both distinctions into account together, that is, inflected vs. derived words and nouns vs. verbs.

Therefore, the present study aims to investigate differences between inflectional and derivational processes in nouns and verbs. More specifically, we used inflected Greek nouns and verbs and we asked whether their lexical processing is differentially facilitated by prior presentation of their stem in words of the same grammatical class (inflectional morphology) or of a different grammatical class (derivational morphology). In other words, we investigated whether grammatical class influences the processing of complex words. If Greek nouns and verbs do not produce equal facilitation to each other (i.e., across grammatical classes: N→V, V→N) as that produced by words of the same grammatical class (i.e., within grammatical class: N→N, V→V), then we might conclude that the root that is common in nouns and verbs has grammatical class-specific representations and, therefore, that grammatical class may constitute part of lexical organization.

**Figure 1:**
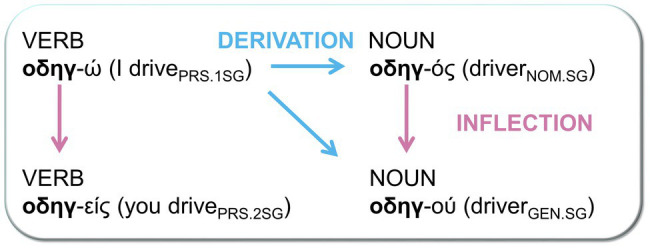
Example of inflectional and derivational process in nouns and verbs using the same stem.

Greek is a highly inflected language that is particularly appropriate for this kind of investigation because of the existence of certain inflectional classes in which the same stem may appear with either verb or noun inflectional suffixes. In these classes, derivation is implied by simply adding verb or noun inflectional suffixes to the same stem. Thus, both inflected and derived words are suffixed, but no derivational affixes are involved. As shown in Figure 1, a stem such as “οδηγ-” can be either a verb or a noun stem, and which one it is depends on whether it is suffixed with a verb or noun inflection. Verb inflectional suffixes such as -ω and -εις indicate number, person, and tense, whereas noun inflectional suffixes such as -oς and -ου indicate number, gender, and case. This permits precise control of orthographic and phonological overlap between inflected and derived word pairs, while minimizing differences in meaning. The lack of derivational affixes also avoids any influence of (affix) productivity differences in morphological processing (see, e.g., Baayen, 1994; Bertram et al., 1999).

Greek is relatively more balanced in the morphological complexity of nominal and verbal inflection than other, more studied, languages. Gender, case, number, and inflectional class are typically reflected in nominal inflections, whereas person, number, tense, mood, and aspect are the main features of verbal inflections (Ralli, 2005). The ratio of verb-to-noun inflected word forms can range between 13:1 and 4:1, depending on inflectional class (see Ralli, 2003, 2005; Klairis and Babiniotis, 2004; Holton et al., 2012; for comprehensive descriptions of the inflectional system). Thus, Greek is more balanced in this respect compared to other highly inflected languages in the literature, such as Hebrew (1:14; Deutsch et al., 1998) and Italian (between 25:1 and 17.5:1; Traficante and Burani, 2003). In combination, these features of Greek morphology minimize the risk of differences between inflection and derivation processes, or between the processing of nouns and verbs, that might be attributed to differences in semantic or syntactic information, or to differences in morphological complexity.

On the one hand, previous studies have been devoted to the contrast between the two types of morphological relations aiming to examine (a) whether their processing relies on similar or distinct morphological mechanisms, and (b) whether semantic similarity can account for differences in morphological processing during lexical processing. Differences in processing of inflectional and derivational morphological relations have been traditionally explained either on the basis of the operation of distinct mechanisms (e.g., Stanners et al., 1979; Pinker, 1991; Taft, 1994, but cf. Fowler et al., 1985; Raveh and Rueckl, 2000; suggesting equal processing between inflections and derivations), or, alternatively, on the basis of other factors that are reflected in inflected vs. derived words, such as frequency, suffix productivity, morphological family size, semantic transparency, regularity, and suffix allomorphy (Caramazza et al., 1988; Pinker, 1991; Frauenfelder and Schreuder, 1992; Schreuder and Baayen, 1995; McQueen and Cutler, 1998; Clahsen et al., 2011). These studies have typically used words of different grammatical classes (adjectives, nouns, and verbs) indiscriminately (e.g., Raveh and Rueckl, 2000), while other studies have used words of a single grammatical class (nouns only; e.g., Álvarez et al., 2011; or nominal stems; Leminen et al., 2011; or only nouns to test derivation and only verbs to test inflection; Prins et al., 2019) when contrasting different types of morphological relation. The study of Whiting et al. (2013) constitutes an exception, in which the grammatical category (bakes vs. beaks; verbs vs. nouns) is considered together with the presence of morphological complexity (affixed vs. non-affixed words) in a spoken word comprehension task contrasting inflectional and derivational processing using combined magneto- and electroencephalography.

On the other hand, previous studies focusing on the distinction between nouns and verbs have aimed to examine whether grammatical class *per se* is an organizing principle of lexical knowledge. In this context, the differential processing observed for nouns and verbs may result from a genuine word class distinction in representation and processing or, alternatively, from semantic differences and distributional cues/patterns of co-occurrences distinguishing nouns from verbs across languages (see Vigliocco et al., 2011, for a review). Methodological differences (e.g., tasks involving sentences vs. single words) and differences in processing demands between nouns and verbs may also be implicated. Moreover, it is not entirely clear when the representation of grammatical category becomes available during lexical processing, an issue linked to the timing of semantic effects during recognition. In examining processing differences between nouns and verbs, one should take into account morphological complexity and how morphology is processed (Yokoyama et al., 2006). Therefore, whether and how grammatical class is represented in the lexical system needs to be addressed by theories of morphological processing of visual word recognition (Crepaldi et al., 2014).

The two distinctions, namely inflection vs. derivation and nouns vs. verbs, are rarely examined together. As both may be largely driven by semantic differences, it is possible that they constitute two versions of a fundamentally similar distinction. Thus, it is important to consider them together with tightly controlled stimuli that allow cross-comparisons while minimizing confounding differences based on syntactic and semantic processes and morphological complexity. In the present exploratory study, inflected Greek noun and verb targets were used to examine whether their lexical processing is facilitated by prior presentation of morphologically related words. In particular, we aimed to examine whether processing is *differentially* facilitated by prior presentation of their stem in words of the same grammatical class (inflectional morphology) or of a different grammatical class (derivational morphology). In this way, we are simultaneously considering effects of the type of morphological relation and grammatical class.

If morphological relations affect processing of complex Greek words, consistent with studies using the same paradigm in different languages (e.g., Forster et al., 1987; Crepaldi et al., 2010), then morphologically related primes should produce facilitation, compared to unrelated primes. If inflection and derivation are categorically different morphological processes, handled differently by the mental lexicon or governed by at least partially distinct neural mechanisms (e.g., Niemi et al., 1994), then this may lead to differential facilitation. For example, if inflected words undergo morphological decomposition during their processing, this may result in larger facilitation from inflected than from derived primes (e.g., Stanners et al., 1979; Klimovich-Gray et al., 2017). Similarly, if grammatical class is represented in a way that can affect visual word identification, for example, if root morphemes shared by nouns and verbs have separate representations (see Wheeldon et al., 2019), then grammatical class should affect the processing of complex words such that nouns and verbs will not produce equal facilitation to each other (i.e., across grammatical classes) as that produced by words of the same lexical category (i.e., within grammatical class).

Importantly, if the distinction between the representation of inflected and derived words is closely related to the distinction between the representation of different classes of words (nouns and verbs), we would expect similar patterns of priming effects between different types of morphological relation and between nouns and verbs, across experiments. This is the main guiding hypothesis driving the current exploratory study.

Alternatively, it may be syntactic/grammatical and semantic processing differences that underlie representation and processing differences between morphological relations (inflected vs. derived) and/or between word classes (nouns vs. verbs; see Amenta and Crepaldi, 2012). In that case, we would expect similar processing for both types of morphological relations and/or both word classes, respectively, because grammatical and semantic differences were minimized in our experiments due to the tight control of stimuli that Greek morphology permits. More specifically, differences based on syntactic/grammatical and semantic processing are generally thought to be unlikely to occur very early in processing. If they exist, they will become more evident when semantics comes into play, at a later stage of lexical processing (e.g., Raveh, 2002, but cf. Feldman et al., 2009, 2015), or when semantic processing demands are maximized either by the task (Experiments 3 and 4) or by properties of the stimuli (Experiment 2).

To sum up, in the following set of exploratory experiments we have set out to determine if there are differences in morphological priming depending on morphological relation (inflection vs. derivation) or word class (noun vs. verb).

## Experiment 1

In the first experiment, we used masked priming to examine whether the processing of Greek noun and verb targets is facilitated when these are preceded by morphologically related prime words, either inflectionally or derivationally related ones, and whether the type of morphological relation modulated the priming effect. Verbs and nouns were primed by words belonging to the same grammatical class (inflectional relation) and by words belonging to a different grammatical class (derivational relation). Thus, we also examined whether grammatical class affects lexical processing.

### Method

#### Participants

Sixty native speakers of Greek (38 females; education *M*=16.15years, SD=2.18) between the ages of 18 and 35years (*Μ*=24.2, SD=3.4) participated in the present study for course credit or volunteered. All participants were students reported having normal or corrected-to-normal vision and no diagnosis of dyslexia or known reading or speech disorders.

#### Materials

Thirty-one Greek word quadruplets were selected. Each quadruplet was formed using a single orthographic stem and four inflectional suffixes, including two verb and two noun suffixes, resulting in two inflectional variants of a verb (V1 and V2; e.g., V1: οδηγεί, /oðiʝi/, “he drives_PRS.3SG_” and V2: οδηγούν, /oðiɣun/, “they drive_PRS.3PL_”) and two inflectional variants of a noun (N1 and N2; e.g., N1: οδηγοί, /oðiʝi/, “drivers_NOM_._PL_” and N2: οδηγού, /oðiɣu/, “driver_GEN_._SG_”). The morphological relationship between verbs and nouns in each quadruplet was verified using the Dictionary of the Modern Greek Language (Babiniotis, 2002). All words were verified to be non-compound words in the dictionaries of the Portal for the Greek Language.^2^

More specifically, the verb inflectional suffixes marked the third person singular present (V1: -α /-a/ and -ει /-i/, 15 and 16 times, respectively) and the third person plural present (V2: -ουν /-un/), while the noun inflectional suffixes marked the nominative plural (N1: -οι, /-i/ (masculine) and -ες, /-es/ (feminine: 14 and 17 times, respectively) and the genitive singular (N2: -ου /-u/ (masculine: 14 times) and -ης /-is/ and -ας /-as/ (feminine: 6 and 11 times, respectively)). These noun and verb inflectional suffixes were selected because they permitted frequency matching between verb and noun pairs. Specifically, there was no significant difference in frequency between V1 (*M*=5.60, SD=16.17) and V2 (*M*=3.88, SD=8.90) pairs, *t*(60)=0.52, *p*=0.606; nor between N1 (*M*=2.23, SD=4.28) and N2 (*M*=6.61, SD=20.17) pairs, *t*(60)=−1.18, *p*=0.242; nor between V1 and N1 pairs, *t*(60)=1.12, *p*=0.267. Suffixes and their frequencies are listed in Table 1. An example of verbs and nouns in a quadruplet is shown in Table 2. Verbs and nouns belonged to the same verb and noun inflectional class, respectively. Verbs were systematically not stressed on the stem, whereas nouns were either stressed on the stem (14 times) or not (17 times).^3^ The full set of stems and test word quadruplets can be found available on OSF.^4^

**Table 1:** Suffixes used in Experiments 1–4 and their frequencies.

V1		V2		N1		N2	
suffix	freq.	suffix	freq.	suffix	freq.	suffix	freq.
-ει /-i/	181.4	-ουν /-un/	77.2	-οι /-i/	48.1	-ου /-u/	28.2
-α /-a/	45.2	-ουν /-un/	28.8	-ες /-es/	99.2	-ας /-as/	67.1
				-ες /-es/	129.3	-ης /-is/	47.2
**Average**	113.3		53		92.2		47.5

*Frequencies (freq.) are specified in occurrences per million tokens*.

**Table 2:** An example of verbs and nouns in a quadruplet of Experiments 1–4.

Root	V1	V2	N1	N2
οδηγ-	οδηγεί	οδηγούν	οδηγοί	οδηγού
drive	drive_PRS.3SG_	drive_PRS.3PL_	driver_NOM.PL_	driver_GEN.SG_

Words in each test quadruplet were matched in (log-transformed) word frequency, number of letters, and number of syllables, based on the “C” corpus of the ILSP Psycholinguistic Resource (IPLR; Protopapas et al., 2012) (see Table 3).^5^ The orthographic and phonological overlap of both nouns and verbs in each quadruplet was held constant, as all stimuli included the same stem and only differed in the inflectional suffix. Eight pairs were phonologically identical and were only distinguished orthographically.

**Table 3:** Stimulus variables for primes and targets of Experiments 1–4.

Variables	Target		Prime							
			Inflection				Derivation			
	**V1**		**V2**		**CV2**		**N1**		**CN1**	
	M	SD	M	SD	M	SD	M	SD	M	SD
Frequency	0.03	0.80	0.03	0.75	0.03	0.76	0.18	0.74	0.10	0.73
N letters	6.20	1.45	7.68	1.35	7.71	1.35	7.03	1.62	7.00	1.65
N syllables	2.68	0.65	2.68	0.65	2.68	0.65	2.87	0.88	2.88	0.89
OLD20	2.16	0.42	2.38	0.33	2.35	0.34	2.24	0.40	2.22	0.40
	**N1**		**N2**		**CN2**		**V1**		**CV1**	
	M	SD	M	SD	M	SD	M	SD	M	SD
Frequency	0.18	0.74	0.00	0.92	0.00	0.92	0.03	0.80	0.02	0.80
N letters	7.03	1.62	7.03	1.62	7.03	1.62	6.20	1.45	6.20	1.45
N syllables	2.87	0.88	2.90	0.87	2.90	0.87	2.68	0.65	2.68	0.65
OLD20	2.24	0.40	2.15	0.43	2.15	0.45	2.16	0.42	2.09	0.37

In each quadruplet, one of the two verb inflectional variants (V1) and one of the two noun inflectional variants (N1) were selected as targets^6^ for the lexical decision task. Each target was paired with morphologically related primes (V1 primed by V2 or N1; N1 primed by N2 or V1), and with unrelated control primes.

Control primes were selected, pairwise matched to the morphologically related primes in number of letters, number of syllables, frequency, and orthographic neighborhood density (Orthographic Levenshtein Distance 20, OLD20; Yarkoni et al., 2008). Specifically, control verbs (CV1 and CV2) were matched to verb primes (V1 and V2, respectively) and control nouns (CN1 and CN2) were matched to noun primes (N1 and N2, respectively). All control primes were orthographically, phonologically, and semantically unrelated to their corresponding targets (V1 and N1).

Consequently, target words (V1 and N1) appeared in five experimental conditions. Verb targets (V1) were primed (a) by morphologically related verbs (V2-V1 condition); (b) by unrelated control verbs (CV2-V1 condition); (c) by morphologically related nouns (N1-V1 condition); (d) by unrelated control nouns (CN1-V1 condition); and (e) by themselves (the Identity condition: V1-V1). Similarly, noun targets (N1) appeared in five corresponding priming conditions: N2-N1; CN2-N1; V1-N1; CV1-N1; and N1-N1.

For both verb and noun targets, prime and target words are of the same grammatical class in Condition 1 (V2-V1 and N2-N1), which therefore concerns inflectional morphology. In contrast, target words and primes are of different grammatical class in Condition 3 (N1-V1 and V1-N1), which therefore concerns derivational morphology. The Identity condition (V1-V1 and N1-N1) was only included to establish a baseline and is not considered further in the present report. Table 4 provides a summary of all experimental conditions with examples.

**Table 4:** Summary of all experimental conditions of Experiments 1–4 with examples.

Target	Prime				
	Inflectional morphology		Derivational morphology		Identity
	Condition 1	Condition 2	Condition 3	Condition 4	Condition 5
**V1**	**V2**	**CV2**	**N1**	**CN1**	**V1**
οδηγεί	οδηγούν	πάρουμε	οδηγοί	ουσίες	οδηγεί
/oðiʝi/	/oðiɣun/	/paroume/	/oðiʝi/	/usies/	/oðiʝi/
drive_PRS.3SG_	drive_PRS.3PL_	take_PRS.1PL_	driver_NOM.PL_	substance_NOM.PL_	drive_PRS.3SG_
**N1**	**N2**	**CN2**	**V1**	**CV1**	**N1**
οδηγοί	οδηγού	ορισμό	οδηγεί	έμεινε	οδηγοί
/oðiʝi/	/oðiɣu/	/orizmo/	/oðiʝi/	/emine/	/oðiʝi/
driver_NOM.PL_	driver_GEN.SG_	definition_ACC.SG_	drive_PRS.3SG_	stay_PRS.3SG.PST._	driver_NOM.PL_

Besides word targets V1 and N1 used in the experimental conditions, an equal number of nonword targets were included. These were created by substituting the first consonant of word targets and were matched to them in length and bigram frequency. Nonword targets were thus composed of meaningless stems and legal inflectional suffixes, resulting in orthographically and phonologically legal letter strings that overlapped in form with word targets. They were paired with real-word primes in a similar fashion as the targets, yielding the same conditions of word prime-nonword target as for word-word pairs. Thus, nonword targets were preceded by unrelated word primes that were matched to test word primes in length, number of syllables, frequency, and orthographic similarity, or by identity primes.

Sixty-four unrelated filler word/word pairs and another 64 unrelated filler word/pseudoword pairs were also included, to reduce the proportion of trials in which primes and targets were morphologically related. This was meant to minimize the direction of attention to the morphological relations. Filler word targets and primes were matched on length and number of syllables to test targets and primes, respectively. Filler pseudowords were orthographically and phonemically regular, generated by changing the first consonant of real words, and were matched on length and number of syllables to both test targets and corresponding filler word primes.

Five experimental lists were created, each containing all of the nonword and filler trials, and all of the noun and verb targets. Each target appeared in each list in a single condition, counterbalanced among lists. The allocation of target conditions to lists was pseudorandomized in Excel prior to data collection. Noun and verb targets were thus presented to each participant only once, while priming conditions were equally distributed among the five lists. Each participant was presented with only one of the lists, and the order of trials was individually randomized.

#### Apparatus and Procedure

Participants were administered a lexical decision task with visual targets. Stimulus presentation and response collection was controlled by DMDX display software (Forster and Forster, 2003). Each trial began with a forward mask consisting of a row of 12 hash marks (#) presented at the center of the screen for 500ms, followed by the prime, which remained on the screen for 50ms (three frames at 60Hz refresh rate) and was immediately replaced by the target, which remained on the screen for 1,000ms or until a response was made for up to 2,000ms. The next trial began 1,500ms after the response (or the expiration of the timeout period). All stimuli were presented in black on a white background and in lowercase so as to include stress diacritics. Primes were displayed in 12-point Arial and targets in 16-point Arial.

Participants responded using two buttons on the computer keyboard, with positive responses given by the dominant hand. They were not informed of the presence of primes. The experimental session consisted of 314 trials and included an equal number of word and pseudoword targets. Their order of presentation was randomized for each participant and split into two blocks separated by a short break. Each session started with a practice block (14 trials) to familiarize participants with the task. Response times (RTs) were measured from the onset of target presentation.

#### Data Analysis

Data were analyzed in R statistical software (R Development Core Team, 2014) with mixed-effects models (Baayen, 2008; Baayen et al., 2008) including crossed random effects of participants and items. Models excluded interactions and correlations of random effects, to allow convergence. Response times were transformed to a logarithmic scale to approximate normality. All analyses were carried out using function lmer of the lme4 package (version 1.1–7; Bates et al., 2014); *p* values were calculated based on the Satterthwaite approximation using package lmerTest (version 2.0–25; Kuznetsova et al., 2014). Following Baayen and Milin (2010), fixed effects of trial order and log response time in the preceding trial were added to the model to account for temporal dependencies; a random slope of trial order was also added to model individual variability in longitudinal effects (such as learning or fatigue). Nominal predictor variables were difference-coded (−0.5 vs. +0.5); numeric predictor variables were centered and scaled to *M*=0 and SD=1.

### Results

Mean response times and error rates per condition are shown in Table 5. In this and the following experiments, a criterion of at least 85% correct responses on experimental items was applied, intending to exclude participants with low performance (possibly inattentive). No participants were excluded in the present experiment.

**Table 5:** Response times and accuracy per condition in Experiment 1.

Prime	Target							
	Inflection				Derivation			
	Noun		Verb		Noun		Verb	
	M	SD	M	SD	M	SD	M	SD
Related	690	133	661	135	710	129	665	122
Unrelated	736	131	700	125	720	124	695	127
Priming Effect	46		39		10		30	
Related	1.1	5.1	2.1	5.4	2.4	5.7	2.4	5.9
Unrelated	1.8	5.1	1.3	4.5	3.6	8.1	3.0	7.1

*Response times are shown in ms at the top of the table; Accuracy is presented in percent error at the bottom of the table*.

An initial omnibus analysis of response times examined the presence of priming effects and their modulation by the type of morphological relationship and by the grammatical class of the target word. Specifically, the model formula included fixed effects of prime relation (Prime: morphologically related vs. unrelated), morphological relationship (Morph: inflection vs. derivation), grammatical class of the target word (Class: Noun vs. Verb), trial order, and log response time in the immediately preceding trial. Prime, Morph, and Class were allowed to interact fully. Random effects included random intercepts for participants and items as well as noninteracting random slopes for Prime, Morph, Class, and trial order, per participant, and for Prime and Morph per item.

**Figure 2:**
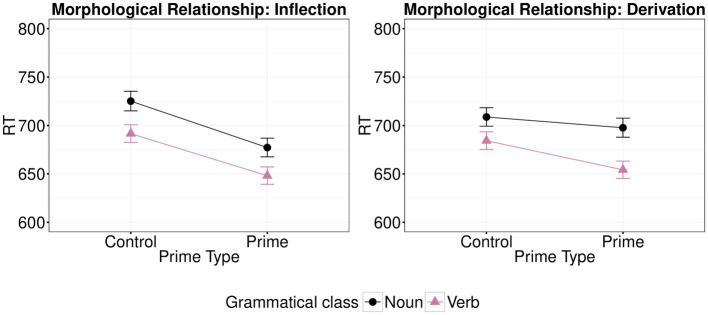
Effects of morphological type (control vs. prime) on mean response times (back-transformed from logRT) for each type of morphological relationship (inflection vs. derivation) and each target (verb vs. noun) in Experiment 1. Error bars show standard error.

The analysis showed a significant main effect of Prime Type (*β*=−4.60, *t*=−6.16; *p*<0.001), of Class of the target word (*β*=−4.48, *t*=−2.07; *p*=0.043), but no significant main effect of Morphological Relationship (*β*=−4.96, *t*=−0.06; *p*=0.954). Class did not interact with either Prime Type (*β*=−1.77, t=−1.19; *p*=0.240) or Morphological Relationship (*β*=−1.37, *t*=−0.08; *p*=0.935). However, there was a significant interaction between Prime Type and Morphological Relationship (*β*=−2.83, *t*=−1.98; *p*=0.048). Thus, the effects of morphological type on priming (shown graphically in Figure 2) were subsequently examined separately for each type of morphological relationship, with both grammatical classes taken together. There was a significant effect of Prime Type for inflection (N2-N1 vs. CN2-N1 and V2-V1 vs. CV2-V1; *β*=−5.95, *t*=−5.45; p<0.001) and for derivation (V1-N1 vs. CV1-N1 and N1-V1 vs. CN1-V1; *β*=−0.03, *t*=−3.09; *p*=0.003). Therefore, there was greater facilitation between morphologically related vs. unrelated primes for inflection than for derivation. This means that there was a greater facilitatory effect when targets were preceded by morphologically related primes when the morphological relationship was an inflection than when it was a derivation (see Figure 2). There was no significant triple interaction of Prime Type, Class and Morphological Relationship (*β*=3.13, *t*=1.10; *p*=0.271). The full list of fixed effects along with the R code used to run the analysis are available on OSF (https://osf.io/4mtzp/).

### Discussion

The results of Experiment 1 indicate that both noun and verb targets are significantly facilitated when primed by morphologically related versus unrelated words. Importantly, differences between inflectionally versus derivationally related word pairs were present with both noun and verb targets, suggesting differences in processing inflectional versus derivational morphological relations. In contrast, no significant differences in priming facilitation were observed for noun vs. verb targets. The fact that priming effects differed between different types of morphological relations but not between complex words of different grammatical class suggests that the distinctions of morphological relation and grammatical class are not manifestations of a single underlying categorical distinction (or two closely related ones).

The differential processing of inflected and derived verbs and nouns was found after only 50ms of morphological prime presentation. This may indicate that morphemic representations are accessed early in visual word processing (e.g., see Rastle and Davis, 2008 for review), and that early effects of semantics may arise during morphological processing (e.g., Feldman et al., 2009). Early morphological processing and the possible contribution of semantic effects are further examined in the following experiments.

In Experiment 1, we randomly selected one of the two verb inflectional variants (V1) and one of the two noun inflectional variants (N1) as targets for the lexical decision task, whereas the other variants (V2, N2) were selected as primes. Because this was a completely arbitrary decision, we decided to test whether the asymmetrical priming between inflectionally vs. derivationally related prime target pairs still emerges with the reversed prime-target selection of Experiment 1, as a replication and effective test of validity of the observed effect. The reversal of prime-target allocation allows us to examine whether differences in priming may be modulated by morphological properties such as inflectional variants, given that differential processing for nominative and non-nominative cases within nouns has been suggested in previous studies (Lukatela et al., 1980; Feldman and Fowler, 1987; Yokoyama et al., 2012). It should be noted, however, that no specific hypothesis was posited prior to conducting the experiment.

## Experiment 2

Experiment 2 further explores the possibility that the differences in morphological priming for inflectionally vs. derivationally related word pairs observed in Experiment 1 may be due to differences in prime-target selection. Thus, we administered the same lexical decision task using masked priming, with the difference that in the present experiment prime target word selection was reversed. If the asymmetry reflected the prime-target word selection, we expected this change in prime-target words to influence the pattern of results, specifically to diminish any asymmetrical priming effects between inflectionally and derivationally related prime target words. Importantly, after reversal of prime-target allocation, verb targets appeared in the third person plural and verb primes in the third person singular, whereas noun targets appeared in the genitive case and noun primes in nominative plural.

### Method

#### Participants

Sixty-three Greek native speakers (39 females, mean years of education: 15.57, SD=1.18) aged 18–35 (*M*=22.2, SD=2.3) similar in characteristics to those of the Experiment 1 (i.e., students, no dyslexia, etc.). None had participated in Experiment 1.

#### Materials, Apparatus, and Procedure

Everything was identical to Experiment 1 except that verb and noun targets of Experiment 1 were presented as primes and vice versa. Specifically, V2 was now primed by V1 or N2, and N2 was primed by N1 or V2. There was again a frequency matching between verb and noun pairs. In addition to the comparisons already reported under Experiment 1, there was no significant differences in frequency between V2 (*M*=3.88, SD=8.90) and N2 (*M*=6.61, SD=20.17) pairs; *t*(60)=1.01; *p*=0.318). There was no change in the nonword and filler trials.

### Results

Mean response times and error rates per condition are shown in Table 6. No participants were excluded.

**Table 6:** Response times (ms; top) and accuracy (percent error; bottom) per condition in Experiment 2.

Prime	Target			
	Inflection				Derivation			
	Noun		Verb		Noun		Verb	
	M	SD	M	SD	M	SD	M	SD
Related	695	123	632	94	711	129	626	73
Unrelated	721	142	667	102	738	146	666	109
Priming Effect	26		35		27		40	
Related	7.0	11.6	1.2	5.0	7.5	10.9	1.0	5.0
Unrelated	5.3	9.8	2.0	5.4	6.7	10.0	1.3	4.4

*Response times are shown in ms at the top of the table; Accuracy is presented in percent error at the bottom of the table*.

**Figure 3:**
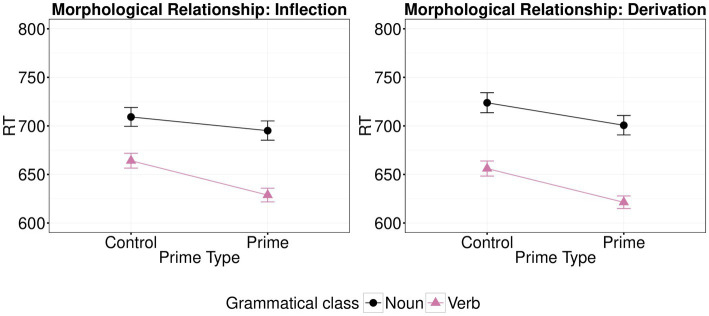
Effects of morphological type (control vs. prime) on mean log-transformed response times (logRT) for each type of morphological relationship (inflection vs. derivation) and each grammatical class (noun; right and verb; left) in Experiment 2. Error bars show standard error.

The effects of morphological type on priming for each type of morphological relationship and each grammatical class are shown graphically in Figure 3. There was a significant main effect of Prime Type (*β*=−4.37, *t*=−6.03; *p*<0.001) and of Class of the target word, with verb targets being faster than noun targets (*β*=−9.73, *t*=−4.41; *p*<0.001), but no significant main effect of morphological relationship (*β*=−9.04, *t*=−1.21; *p*=0.231). As in Experiment 1, Class did not interact with either Prime Type (*β*=−2.31, *t*=−1.59; *p*=0.111) or Morphological Relationship (*β*=2.64, *t*=1.83; *p*=0.067). Moreover, there was no significant interaction between Prime Type and Morphological Relationship (*β*=9.37, *t*=0.07; *p*=0.948). Although technically not required, given the nonsignificant interaction, simpler models were employed for direct comparison with Experiment 1. Significant facilitation between morphologically related vs. unrelated primes was observed for both inflection (V2-V1 vs. CV2-V1 and N2-N1 vs. CN2-N1; *β*=−4.37, *t*=−4.34; *p*<0.001) and derivation (N1-V1 vs. CN1-V1 and V1-N1 vs. CV1-N1; *β*=−4.49, *t*=−3.99; *p*<0.001). Also, there was no significant interaction of Class, Prime Type and Morphological Relationship (*β*=−1.05, *t*=−0.04; *p*=0.971). The full list of fixed effects along with the R code used to run the analysis are available on OSF.

### Discussion

Consistent with the results of Experiment 1, there was facilitation in processing both noun and verb targets when preceded by morphologically related words. However, unlike Experiment 1, there was no difference between inflectional and derivational processing or, in other words, when words of the same or different grammatical class preceded the target, when the latter was either a noun or a verb. Thus, no asymmetrical priming facilitation between inflectionally vs. derivationally related pairs was observed when we reversed prime-target selection. Taking into account the differences in target stimuli between Experiments 1 and 2, we may attribute the absence of asymmetrical priming to the case change in noun targets to a non-nominative case, which may have increased the processing demands of the task. In other words, we suggest that increased processing demands of noun targets diminished priming facilitation to these targets and thereby reduced the asymmetry in priming effects, relative to Experiment 1. The apparent influence of increased processing demands with short prime presentation is consistent with an early role of semantic information in early morphological processing (e.g., Feldman et al., 2009).

In fact, noun targets yielded significantly longer decision times than verb targets in the present experiment, consistent with this interpretation. This finding is also consistent with earlier suggestions that oblique cases yield longer decision times than the nominative and, consequently, that the lexical organization within nouns may differ for nominative and non-nominative cases (Lukatela et al., 1980; Feldman and Fowler, 1987), and with a more recent suggestion that there are differences in case processing, and specifically differences in structural complexity between genitive and nominative cases, that induce different brain activations (Yokoyama et al., 2012). Moreover, in Greek, the presentation of a single word in the genitive case is atypical, as the genitive case always depends on another word, such as a verb, a noun, a pronoun, a numeral, or it is a complement of a preposition, an adjective, an adverbial, etc. (Mackridge, 1985; Theofanopoulou-Kontou, 1989), further strengthening the interpretation of differential case processing of the genitive (vs. the nominative) case.

Importantly, in Εxperiments 1 and 2, facilitation was found after 50ms of morphological prime presentation, indicating that morphemic representations are accessed early in visual word processing, consistent with previous studies using the masked priming paradigm. Using this short prime duration, we have observed differences between morphological relations (Experiment 1) and between nominative and non-nominative cases (Experiment 2), consistent with the idea that morphological facilitation is modulated by semantic effects (Experiment 1) and/or processing demands (Experiment 2). However, at such early stages of processing, some studies also report early morphological facilitation even for words that are pseudo-morphologically related (e.g., corner-CORN) proposing an early morpho-orthographic stage in visual word processing that operates independently of semantic relationship (Marslen-Wilson et al., 1994; Rueckl and Raveh, 1999; Plaut and Gonnerman, 2000; Giraudo and Grainger, 2001, 2003; Feldman et al., 2004; Lázaro et al., 2016; Heyer and Kornishova, 2018).^7^ This raises the question of whether it is truly semantic effects we are observing.

To further explore the asymmetrical priming and its potential modulation by (early) semantic effects, we examined whether priming effects would be strengthened when primes are fully visible, in a task that is thought to favor semantic effects (Experiment 3), and maintained when primes are fully visible and multiple items intervene between primes and targets, in a task that is more dependent on morphological relations (Experiment 4). Using paradigms that are considered to be less sensitive to orthographic overlap also allowed us to (indirectly) explore whether pure morphological relatedness between pairs – and not orthographic overlap – could account for the priming facilitation produced at early stages of processing. Thus, in the following experiments, we explored priming effects in paradigms reflecting later morpho-semantic processing stages in visual word recognition (Orfanidou et al., 2011).

## Experiment 3

In Experiments 1 and 2, morphological priming effects were observed with a very short prime duration (50ms), indicating that morphemic representations appear to be available in early stages of visual word recognition. At that early point in processing, differences between inflection and derivation processes (Experiment 1), as well as differences in processing nouns in nominative vs. non-nominative cases (Experiment 2), were present, indicating that early semantic effects may also arise and thus modulate morphological facilitation. Therefore, Experiment 3 explores the possibility that semantic effects were underlying the observed asymmetry in priming, thereby obscuring the distinction between morphological relations. In particular, we asked whether priming asymmetry increases at longer prime exposures (250ms), when semantic effects have presumably become more evident (e.g., Feldman and Prostko, 2002; Royle et al., 2019).

Furthermore, Experiment 3 indirectly addresses another possibility, namely that the observed morphological priming effects might be due to the orthographic overlap between prime-target pairs. This is important because it has been suggested that priming effects are also observed for pseudo-morphologically related pairs (e.g., corner-CORN) early in recognition, indicating that orthography plays a crucial role in morphological processing independent of meaning (Marslen-Wilson et al., 1994; Rueckl and Raveh, 1999; Plaut and Gonnerman, 2000; Giraudo and Grainger, 2001, 2003; Feldman et al., 2004; Lázaro et al., 2016; Heyer and Kornishova, 2018). Therefore, we asked whether morphological priming effects are still present at a longer prime duration (250ms). When additional time is available to process the prime, orthographic effects are precluded (they tend toward inhibition; Rastle et al., 2000). Thus, if morphological decomposition is maintained later in the time course of visual word recognition, we could conclude that any priming effects observed would not be attributed to the orthographic relation between prime-target pairs, but to their morphological (or morphological plus semantical) relation.

### Method

#### Participants

Seventy-two Greek students (47 females, mean age: 21.7, SD=1.8 and mean education: 15.22years, SD=1.18) at the University of Athens participated in the experiment in exchange for course credit. The selection criteria for participants were similar as in our previous experiments. None had participated in the previous experiments.

#### Materials, Apparatus, and Procedure

Everything was identical to Experiment 1 except that primes remained on the screen for 250ms (15 frames).

### Results

Mean response times and error rates per condition are shown in Table 7. No participants were excluded.

**Table 7:** Response times (ms; top) and accuracy (percent error; bottom) per condition in Experiment 3.

Prime	Target							
	Inflection				Derivation			
	Noun		Verb		Noun		Verb	
	M	SD	M	SD	M	SD	M	SD
Related	687	110	633	114	731	160	675	121
Unrelated	734	129	719	119	728	133	703	102
Priming Effect	47		86		-3		28	
Related	2.7	8.2	2.0	6.1	4.7	9.4	1.6	7.3
Unrelated	3.0	6.6	3.8	9.0	4.4	10.2	3.8	8.5

*Response times are shown in ms at the top of the table; Accuracy is presented in percent error at the bottom of the table*.

**Figure 4:**
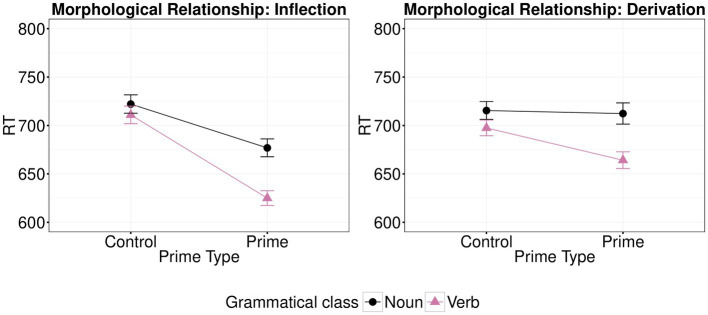
Effects of morphological type (control vs. prime) on mean log-transformed response times (logRT) for each type of target (verb vs. noun) and each type of morphological relationship (inflection vs. derivation) in Experiment 3. Error bars show standard error.

The analysis showed a significant main effect of Prime Type (*β*=−6.43, *t*=−6.22; *p*<0.001) and of Class of the target word (*β*=−4.91, *t*=−2.54; *p*=0.013), but no significant main effect of Morphological Relationship (*β*=−1.79, *t*=−1.91; *p*=0.061). Class did not interact with Morphological Relationship (*β*=9.75, *t*=0.05; *p*=0.959), but the interaction between Class of the target word (Noun vs. Verb) and Prime Type (morphologically related vs. unrelated prime) was significant (*β*=−5.60, *t*=−3. 16; *p*=0.003); thus, we examined simple effects, separately for each Class of the target word. Greater facilitation was shown between morphologically related vs. unrelated primes for verb targets (*β*=−9.13, *t*=−7.46; *p*<0.001) than for noun targets (*β*=−3.80, *t*=−2.44; *p*=0.021). Moreover, the interaction between Prime Type (morphologically related vs. unrelated prime) and type of morphological relationship (inflection vs. derivation) was significant (*β*=−7.28, *t*=−5.03; *p*<0.001). Thus, the effects of morphological type on priming (shown graphically in Figure 4) were examined separately for each morphological relationship. There was greater facilitation between morphologically related vs. unrelated primes for inflection (V2-V1 vs. CV2-V1 and N2-N1 vs. CN2-N1; *β*=−9.84, *t*=−6.59; *p*<0.001), than for derivation (N1-V1 vs. CN1-V1 and V1-N1 vs. CV1-N1; *β*=−2.78, *t*=−2.11; *p*=0.040). There was no significant triple interaction of Prime Type, Class and Morphological Relationship (*β*=−2.19, *t*=−0.76; *p*=0.449). The full list of fixed effects along with the R code used to run the analysis are available on OSF.

### Discussion

Consistent with Experiment 1, the results of the present experiment showed significant facilitatory priming effects to both verb and noun targets when preceded by morphologically related versus unrelated words. Moreover, stronger facilitatory effects were present when both verb and noun targets were preceded by inflectionally rather than derivationally related primes, consistent with the distinction between the representation of inflected and derived words, which here becomes more evident as semantic effects are maximized due to the paradigm used.

The difference in priming facilitation between inflectionally (V-V and N-N pairs) and derivationally related prime-target words (N-V and V-N pairs) was also present at a short prime duration of 50ms (Experiment 1). Replication of this asymmetry in the present experiment corroborates the findings of Experiment 1 and is consistent with the interpretation given in Experiment 2, attributing the absence of asymmetry to the case of target nouns.

Furthermore, the fact that morphological priming effects were still observed with a long prime duration indicates that morphological information is available late in the course of visual word recognition. Thus, priming effects seem not to be triggered by the form overlap between prime target pairs. In other words, Greek readers’ processing of morphemes seems not to be bound to the processing of their orthographic properties. Examining whether morphological effects are still observed using longer SOAs is thus an indirect way to address the possible role of orthographic effects in early morphological processing of Greek complex words. This issue can be further addressed directly in the future by directly comparing the early effects of orthographically related primes with those of morphologically related primes (e.g., Feldman, 2000).

In the present experiment, unlike Experiment 1, facilitation was greater for verb than noun targets. We hypothesize that there may be an inherent asymmetry between noun and verb roots even though the same root was used to form both nouns and verbs. Specifically, according to morphological analysis, the noun stimuli in our experiments were derived by their verb counterparts (Ralli, 2005), even though they both have the exact same stem and no derivational affixes are involved. This means that there may be a more central representation of the root as a verb root than as a noun root, accounting for the greater facilitation when verbs serve as targets as compared to nouns. An alternative explanation might invoke differences in processing demands between these nouns and verbs (Siri et al., 2008). Specifically, processing demands were greater for nouns than for verbs because they were morphologically more complex, being derived by the verbs (Ralli, 2005). That verbs were generally processed faster than nouns was also evident from the comparison of priming effects between verbs and nouns in identity vs. morphological conditions (with identity as a reference level; see on OSF for the analysis script, full output and graphs), which could be explained either in terms of a verb- vs. noun- centrality hypothesis or, alternatively, in terms of reduced processing demands for verbs and/or greater for nouns. The asymmetry is discussed further in the section General Discussion.

## Experiment 4

In the present experiment, we used a long-lag priming experiment to further test whether the pattern of results observed in Experiment 1 is preserved not only when the prime is fully visible but also when the interval between prime and target is long and occupied by unrelated words. Long-lag priming paradigms have been suggested to reflect semantic rather than orthographic levels of processing (Orfanidou et al., 2011). Importantly, when items intervene between prime-target pairs in long-lag paradigms, priming effects persist for prime-target pairs that are morphologically or morphologically plus semantically related, whereas they disappear when they are only semantically related (Bentin and Feldman, 1990; Feldman, 2000; Lahiri and Reetz, 2010; Schuster and Lahiri, 2019) or only orthographically related (Orfanidou et al., 2011). Thus, the effects observed between morpho-semantically related prime and target words in these paradigms rely more on the morphological relation between word pairs than on their semantic or orthographic relation.

We hypothesized that if priming effects observed in Experiment 3 reflected semantic relations we would expect the effects to diminish in a task less dependent on semantics. Moreover, if the effects observed in the previous experiments were due to orthographic similarity rather than to morphological relatedness then these effects would also diminish in a task not sensitive to orthographic similarity. However, if priming effects persist, that would be consistent with recognition processes involving morphological information.

### Method

Unlike masked priming (Experiments 1 and 2), in a long-lag priming experiment, participants typically make a lexical decision to both primes and targets. If we used the same stimuli as in Experiments 1 and 2, this would result in doubling the duration of the experimental procedure. To avoid this, we made changes in the filler materials, as described below. The critical prime-target pairs remained exactly the same as in the preceding experiments.

#### Participants

Eighty-three Greek students (40 females, mean age: 22.0, SD: 3.4 and mean education: 15.60years, SD=1.64) at the University of Athens participated in the experiment in exchange for course credit. The selection criteria for participants were similar as in our previous experiments. None had participated in previous experiments.

We used the same 31 word quadruplets and experimental conditions as in Experiment 1. Unlike Experiment 1, nonword targets orthographically related to word targets were not included. Instead, an augmented set of 186 filler pseudowords were used to increase the proportion of “No” responses in the experiment. All were created by changing a letter of an existing word not used in the experiment. Pseudoword targets were preceded by pseudoword primes that were either orthographically related or not. Orthographically related pseudoword primes differed from pseudoword targets only in the suffixes and were matched to the word/word pairs on prime/target form overlap and length. Orthographically unrelated pseudoword primes were generated by changing one consonant letter in the root of a filler word from Experiment 1 and were equated in length to pseudoword targets and to orthographically related primes.

Five experimental lists were created, each containing all of the filler pseudoword trials and all of the noun and verb targets, each in a single condition, counterbalanced among lists. Priming conditions were equally distributed among the five lists. In each list, participants made 372 lexical decisions (186 in response to the prime-target pairs and 186 to the filler pseudoword prime-target pairs). Primes and targets were separated by an average of 12 intervening items (lags ranged from 8 to 16 intervening items).

#### Apparatus and Procedure

Each stimulus was displayed in the middle of the screen and remained until the participant made a response, for up to 1,000ms. Participants had 2,500ms to respond. The intertrial interval was 1,000ms. All stimuli were presented in 16-point Arial.

### Results

Mean response times and error rates per condition are shown in Table 8. No participants were excluded.

**Table 8:** Response times (ms; top) and accuracy (percent error; bottom) per condition in Experiment 4.

Prime	Target							
	Inflection				Derivation			
	Noun		Verb		Noun		Verb	
	M	SD	M	SD	M	SD	M	SD
Related	729	148	680	137	698	144	690	126
Unrelated	782	162	736	150	745	144	756	150
Priming Effect	53		56		47		66	
Related	5.9	8.6	2.1	6.3	3.5	6.3	2.9	9.7
Unrelated	7.6	10.7	4.2	8.7	6.9	8.6	8.1	9.1

*Response times are shown in ms at the top of the table; Accuracy is presented in percent error at the bottom of the table*.

**Figure 5:**
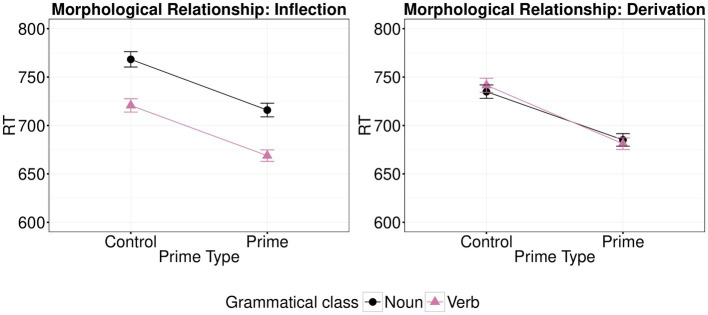
Effects of morphological type (control vs. prime) on mean log-transformed response times (logRT) for each type of morphological relationship (inflection vs. derivation) in Experiment 4. Error bars show standard error.

Results show main effects of Prime Type (*β*=−7.66, *t*=−9.12; *p*<0.001), but no main effects of Class of the target word (*β*=−3.07, *t*=−1.64; *p*=0.106), or Morphological Relationship (*β*=1.00, *t*=1.49; *p*=0.142). Class did not interact with Prime Type (*β*=−1.44, *t*=−1.04; *p*=0.302); however, it significantly interacted with Morphological Relationship (*β*=−6.77, *t*=−5.04; *p*<0.001). Simpler models were employed showing facilitation between inflection vs. derivation for both Nouns (*β*=4.39, *t*=3.82; *p*<0.001) and Verbs (*β*=−2.24, *t*=−2.80; *p*=0.009). The interaction between Prime Type (morphologically related vs. unrelated prime), and type of morphological relationship (inflection vs. derivation) was not significant (*β*=9.34, *t*=0.89; *p*=0.375). The effects of morphological type on priming for each Class and each type of morphological relationship are shown graphically in Figure 5. Simpler models were employed, for direct comparison with the previous experiments. Facilitation between morphologically related vs. unrelated primes was observed for both inflection (V2-V1 vs. CV2-V1 and N2-N1 vs. CN2-N1; *β*=−7.16, *t*=−6.60; *p*<0.001) and derivation (N1-V1 vs. CN1-V1 and V1-N1 vs. CV1-N1; *β*=−8.17, *t*=−8.42; *p*<0.001). Moreover, there was no significant interaction of Class, Prime Type, and Morphological Relationship (*β*=1.52, *t*=0.72; *p*=0.470). The full list of fixed effects along with the R code used to run the analysis are available on OSF.

### Discussion

The results of Experiment 4 showed facilitatory effects to both verb and noun targets when preceded by morphologically related vs. unrelated words. Consistent with the results obtained in Experiment 1, priming effects were still present in a task that is not sensitive to orthographic similarity, indirectly supporting the conclusion that the effects observed in Experiment 1 were not due to form overlap between morphologically related pairs but to their morphological relation, which is consistent with recognition processes involving morphological information.

Unlike Experiment 3, priming effects were of similar magnitude when target words were preceded either by inflectionally or by derivationally related primes. In other words, processing differences were equally observed in the target words when preceded by words of the same versus different grammatical class. This is consistent with the proposal for delayed priming experiments that some activation due to shared semantics might occur for all primes, but it disappears over the delay between prime and target words (Bozic et al., 2007; Wheeldon et al., 2019), and also consistent with our interpretation that priming effects reflect semantic relations.

## General Discussion

The present study examined differences in the magnitude of morphological priming effects induced by inflectionally vs. derivationally related Greek nouns and verbs. Such differences have often been interpreted as evidence for different types of representation and processing between inflectional vs. derivational morphological relations (Stanners et al., 1979; Friederici et al., 1989; Laudanna et al., 1992; Feldman, 1994; Marslen-Wilson et al., 1994; Marslen-Wilson, 2007; Klimovich-Gray et al., 2017) and hence, as an argument against a fully decompositional view of processing morphologically complex word within and across languages (Caramazza et al., 1988; Marslen-Wilson et al., 1994; Schreuder and Baayen, 1995; Baayen et al., 1997). Moreover, these differences have also been interpreted as evidence that there are grammatical class-specific representations, distinct for verbs and nouns, and hence, that the same root morphemes shared by nouns and verbs are marked for grammatical class (this is true at least for some base forms; Wheeldon et al., 2019).

The present study simultaneously examined both differences together, namely between inflected vs. derived words and between nouns vs. verbs, in the Greek language. Greek is a highly inflected language in which words typically consist of a stable stem and verb or noun inflectional suffixes. This permits comparisons between morphologically complex words that do not differ in morphological complexity (cf. English: walk-er; walk-∅). Moreover, because of the existence of certain inflectional classes in which the same stem may appear with either verb or noun inflectional suffixes, both inflection and derivation can be formed using only inflectional suffixes, so that the morphological structure remains transparent. Focusing on this particular inflectional class permits precise control of orthographic and phonological overlap between inflected and derived word pairs, and minimizes or avoids confounding factors that may affect the detection of morphological structure, most notably, semantic transparency and affix productivity. Greek is also relatively more balanced in the morphological complexity of nominal and verbal inflection than other, more studied, languages (e.g., Hebrew; Deutsch et al., 1998; Italian; Traficante and Burani, 2003). In combination, these features of Greek morphology minimize the risk of differences between inflection and derivation processes, or between the processing of nouns and verbs, that might be attributed to differences in semantic or syntactic information, or to differences in morphological complexity and thus permits an unprecedented level of comparability across morphological processes and grammatical classes.

In the present experiments, the pattern of facilitation in lexical decision latencies for target words preceded by morphologically related vs. unrelated primes indicates that Greek readers are sensitive to the morphological structure of morphologically complex Greek words. This facilitation is consistent with the results of other studies using the same paradigm in different languages (Forster et al., 1987; Grainger et al., 1991; Marslen-Wilson et al., 1994; Frost et al., 1997; Rastle et al., 2000; Gonnerman et al., 2007; Crepaldi et al., 2010) and provides evidence for morpheme-based representations in the Greek lexicon. As noted, the fact that Greek permits the exact same stem in certain verb and noun classes (see also zero-derivations in English: Pliatsikas et al., 2014; Wheeldon et al., 2019), in conjunction with the similar size and structure of verb and noun inflectional endings, has allowed us an unprecedented level of stimulus matching across grammatical categories. This lends further support to findings from other languages, in which unavoidable differences, such as additional suffixes, may have diminished the comparability of findings across grammatical classes and morphological processes.

In Experiments 1 and 2, facilitation between morphologically related word pairs is detectable after only 50ms of prime presentation, indicating that morpheme-based representations are accessed early in visual word processing. Morphological priming was also present under longer prime durations, in Experiments 3 and 4. In these experiments, primes and targets were both presented overtly, either in immediate succession (Experiment 3) or with an average lag of 12 intervening items (Experiment 4).

In two of the four experiments, inflectionally related primes produced significantly greater effects than derivationally related primes. Specifically, mean priming facilitation (across verbs and nouns) from inflected primes was 85ms in Experiment 1 and 133ms in Experiment 3; in comparison, priming facilitation from derived primes was 40ms and 25ms, respectively. This suggests that there may be differences in processing inflectional versus derivational morphological relations (e.g., Feldman, 1994). Thus, priming facilitation was sensitive to type of morphological relation (inflection, derivation) even when there were minimal phonological, orthographic, and semantic differences between prime target pairs.

In contrast, no differences between inflected and derived forms were observed under the long-lag priming paradigm (Experiment 4), where facilitation from inflected and derived primes was 109ms and 113ms, respectively. Long-lag paradigms with a lag of intervening items between morpho-semantically related prime-target words are less dependent on semantic relations compared to tasks with no intervening items (Experiment 3; e.g., Bentin and Feldman, 1990; see also Bozic et al., 2007; Wheeldon et al., 2019). This indicates that processing differences between inflected and derived forms diminished when processing was less dependent on semantic effects. In the present study, semantic effects were minimized using inflected Greek nouns and verbs that consisted of the same stem and noun or verb inflectional suffixes. However, this does not preclude that any differences in magnitude of facilitation observed between inflectionally vs. derivationally related prime-target pairs could be attributed to greater semantic overlap between inflectional vs. derivational forms and not to the different representation and processing between them (cf. Stanners et al., 1979; Friederici et al., 1989; Laudanna et al., 1992; Feldman, 1994; Marslen-Wilson et al., 1994; Marslen-Wilson, 2007; Klimovich-Gray et al., 2017).

Asymmetrical priming facilitation between inflectionally vs. derivationally related pairs was absent in Experiment 2 (61 and 67ms, respectively). We speculated that this might be due to the case change in noun targets and the resulting increase in processing demands for nouns vs. verbs. According to Ralli (2005), the nouns of the present study were all derived from the corresponding verbs. Processing demands may have been greater for nouns than for verbs because nouns were morphologically more complex, being derived from verbs (cf. Siri et al., 2008). In this experiment, noun targets were presented in a non-nominative case, specifically, in the genitive. Processing differences among cases have been proposed, such that decision times for a noun in non-nominative cases (such as genitive) are longer than decision times for the same noun in nominative ones (Lukatela et al., 1980; Feldman and Fowler, 1987). Thus, nouns in non-nominative cases are even more complex (Lukatela et al., 1980; Feldman and Fowler, 1987; Yokoyama et al., 2012) and increase processing demands. This could explain the diminished priming facilitation to noun targets being presented in the genitive, compared to the facilitation observed to noun targets being presented in the nominative (Experiment 1), and thus the absence of asymmetrical priming effects between inflectional vs. derivational forms (cf. Wheeldon et al., 2019, reporting absence of underlying morphological complexity effects in early morphological processing).

Differences between inflectionally vs. derivationally related words have also been interpreted as evidence that there are grammatical class-specific representations, distinct for verbs and nouns, and hence that the same root morphemes subserving the formation of both nouns and verbs are marked for grammatical class (see Vigliocco et al., 2011, for a review). In the present study, statistically indistinguishable facilitation effects (across inflection and derivation) were observed to the two word classes (nouns and verbs) in Experiments 1, 2, and 4. Specifically, priming effects to nouns vs. verbs were 56 vs. 69ms (Exp. 1), 53 vs. 75ms (Exp. 2), and 100 vs. 122ms (Exp. 4); in each case, the priming effect for verbs was numerically (but not significantly) larger. Priming effects were significantly stronger for verbs (114ms) than for nouns (44ms) only in Experiment 3. This pattern of facilitation is inconsistent with the one observed for different types of morphological relations (inflection vs. derivation), suggesting that processing of inflectional and derivational forms differs in nouns and verbs. This finding rules out the possibility that differences between inflection and derivation processes and differences between nouns and verbs reflect the same or closely related categorical distinctions. The special importance of this finding is that it arises in a single study that systematically considers both distinctions together in a design that controls for confounding factors.

Differences between nouns and verbs in the present study can be attributed to different processing demands (see Vigliocco et al.’s, 2011, suggestion that grammatical class effects increase as processing demands are increased by tasks and languages). As stated earlier, the nouns of the present study were all derived from the corresponding verbs (Ralli, 2005). This means that, although the same root is used to form both nouns and verbs, there may be a more central representation of this root as a verb root than as a noun root (cf. Vigliocco et al., 2011), accounting for the weaker priming effects observed to noun targets. As an alternative to representational centrality, processing demands may have been greater for nouns than for verbs because nouns were morphologically more complex being derived from verbs (cf. Siri et al., 2008). These hypotheses remain to be further investigated in follow-up research systematically contrasting verbs derived from nouns against nouns derived from verbs, expecting an asymmetry in the opposite direction to emerge for derivationally related words (verbs derived by nouns vs. nouns derived by verbs). Moreover, because nouns in non-nominative cases are even more complex (Lukatela et al., 1980; Feldman and Fowler, 1987; Yokoyama et al., 2012), this could also explain why weaker facilitation is observed when nouns (vs. verbs) serve as targets (Experiment 2). However, facilitatory effects were stronger for verbs when semantic effects were maximized in Experiment 3, suggesting that semantic effects may account for differences in processing between words from different grammatical classes (see also Leinonen et al., 2008; Álvarez et al., 2011), although these were minimized to a great extent in the present study.

Regarding the visual word identification system, findings from the present study in morphologically complex Greek words suggest that morphemic analysis is involved in the identification of words, indeed they point to a system with a direct identification process (cf. Giraudo and Grainger, 2001, 2003; Rastle et al., 2004; Järvikivi et al., 2009; Taft and Nguyen-Hoan, 2010). More specifically, regarding processing of inflected vs. derived words or of words from different grammatical classes (nouns vs. verbs), the present study does not suggest that the visual word identification system processes them in the same way (cf. Caramazza et al., 1988; Marslen-Wilson et al., 1994; Schreuder and Baayen, 1995; Baayen et al., 1997). Rather, it may indicate that some processing steps are common to these types of complex words at early stages of processing, as differences in their processing are not evident when processing is less dependent on semantic effects. Their processing begins to differ especially at later stages of lexical processing, when semantics is more likely to come into play (see also Leinonen et al., 2008; Álvarez et al., 2011).

Although semantic relations seem to (strongly) influence morphological priming at longer prime durations, hence at later stages in visual word recognition (e.g., morpho-semantic decomposition; Marslen-Wilson et al., 1994; Rueckl and Raveh, 1999; Plaut and Gonnerman, 2000; Giraudo and Grainger, 2001, 2003; Feldman et al., 2004), they also seem to arise early in morphological processing (e.g., Feldman et al., 2009). However, at that early stages in processing, some studies using the masked priming paradigm have observed facilitation even for word targets that are pseudo-morphologically related to their primes (e.g., corner-CORN), leading to the proposal that there may be a morpho-orthographic decomposition process that applies at early stages of visual word processing. This decomposition is achieved on the basis of orthographic information independently of any semantic relationship (Taft, 1994; Rastle and Davis, 2003, 2008; Rastle et al., 2004). Therefore, firm conclusions on the influence of semantic information at early phases of morphological processing will rest on evidence against an orthographic overlap account. We indirectly examined the role of orthographic information at early stages in processing, using paradigms that are not thought to reflect orthographic relations (Experiments 3 and 4). Morphological effects were found to be preserved under these conditions (e.g., Feldman, 2000), arguing against an orthographic overlap account for the observed priming. The independent role of orthography and/or semantics in the processing of morphemes needs to be further investigated in a follow-up examination of the distinct or joint effects of orthographic and semantic similarity at early and late stages in processing morphologically complex Greek words during visual word recognition, systematically differentiating between the two dimensions of similarity (orthographic and semantic) and morphological similarity that reflects both shared form and meaning.

In summary, we have tested inflectionally and derivationally related prime-target pairs in a lexical decision task using masked priming (with both short and long stimulus onset asynchrony) and long-lag priming. Our results indicate that morphological relations affect processing of morphologically complex Greek verbs and nouns or, in other words, that Greek readers are sensitive to the morphological structure of these morphologically complex words. They provide support for the existence of differences in processing between inflectionally and derivationally related word pairs in Greek, even when phonological, orthographic, and semantic differences between word pairs are minimized. Importantly, these differences could not reflect the extent of orthographic overlap between pairs, as they were preserved under longer prime duration, when orthographic effects diminish. However, differences in processing inflectional and derivational processes disappear when processing is less dependent on semantic effects or when processing demands increase. Moreover, long-lag priming differed between verbs and nouns, indicating that differences in processing complex words of different grammatical class (nouns and verbs) may be explained, at least to a certain extent, in terms of semantic effects.

It should be noted that our conclusions are only based on 10 suffixes that were particularly selected because they allowed us to examine possible differences in processing words of same or different grammatical class (nouns and verbs), avoiding differences – such as additional suffixes – that may modulate recognition processes and thus generate differential priming effects. To achieve tight control of orthographic and phonological overlap, and to minimize differences in meaning between inflected and derived word pairs, we limited our study in the selection of a restricted number of suffixes. Therefore, our study is more exploratory than explanatory or conclusive for the entire language. Furthermore, our study exclusively focuses on the comparison across morphological processes and only two grammatical classes (nouns and verbs). We restricted our selection in nouns and verbs, because we hypothesized a similar pattern in representation and processing between them and inflected and derived words, and also because they constitute main grammatical categories, typically present in almost all languages, and the issue of the distinction between them in the lexicon along with the questions of whether and to what extent their grammatical properties play a role in organizing the lexical knowledge, are investigated and debated in linguistics, cognitive psychology, cognitive neuropsychology, and neuroimaging. Importantly, the representation of different grammatical classes and different morphological relations that were investigated in the present study using the Greek language also constitutes issues of general interest in other languages. However, the extent to which our findings reflect only the specific characteristics of Greek or can actually be extended to other languages requires further investigation in other languages.

Finally, we must acknowledge that differences in effects between experiments (e.g., Experiments 1 and 2) need not necessarily reflect true underlying differences but may indicate an impact of random sampling, especially given the limited number of participants, necessitating replication prior to drawing final conclusions.

Future research should consider (a) the extent to which there are specialized processes for words from different grammatical classes (nouns and verbs), using words with contrastive asymmetry (nouns derived by verbs vs. verbs derived by nouns); (b) whether asymmetric priming could be due to the engagement of different morpho-syntactic processes or due to differences in processing demands, extending our present findings using more suffixes and/or words of other grammatical classes; and (c) whether there are explicit morphological processes and representations in the visual word recognition system at early and late stages in processing morphologically complex Greek words, beyond form and meaning overlap.

## Data Availability Statement

The raw data supporting the conclusions of this article will be made available by the authors, without undue reservation.

## Ethics Statement

Ethical review and approval was not required for the study on human participants in accordance with the local legislation and institutional requirements. The patients/participants provided their written informed consent to participate in this study.

## Author Contributions

All authors jointly designed the study. SL created the study materials, collected the data, and analyzed the data, supervised by AP. SL drafted the manuscript. All authors contributed to revising the manuscript and approved the submitted version for publication.

## Funding

This research has been co-financed by the European Union (European Social Fund) and Greek national funds through the Operational Program “Education and Lifelong Learning” of the National Strategic Reference Framework – Research Program: THALIS-UOA-COGMEK (project 892, MIS 375737).

## Conflict of Interest

The authors declare that the research was conducted in the absence of any commercial or financial relationships that could be construed as a potential conflict of interest.

## Publisher’s Note

All claims expressed in this article are solely those of the authors and do not necessarily represent those of their affiliated organizations, or those of the publisher, the editors and the reviewers. Any product that may be evaluated in this article, or claim that may be made by its manufacturer, is not guaranteed or endorsed by the publisher.

## Footnotes

^1^For a discussion on the key role of semantic compositionality and affix productivity on representation of derived words at the early stages of visual word recognition across languages: English: Feldman et al., 2004; Italian: Carota et al., 2016; Polish: Reid and Marslen-Wilson, 2003; Bozic et al., 2013; but *cf*. German: Smolka et al., 2019; Semitic languages: Boudelaa and Marslen-Wilson, 2004, 2015; Ussishkin et al., 2015.

^2^ http://www.greek-language.gr/greekLang

^3^Stress pattern differences between inflectionally vs. derivationally related pairs that follow from the selected materials used in the present study could not affect visual target word recognition in Greek. This was based on the absence of stress priming effects to visual target words in stress priming experiments in Greek (e.g., Protopapas et al., 2016), which is consistent with the proposal that abstract stress representations are not activated during visual word processing and that stress information is primarily lexical, and argues that Greek readers do not rely entirely on the stress diacritic despite its reliable presence and its excellent cue validity (Protopapas, 2016).

^4^ https://osf.io/4mtzp/

^5^ http://speech.ilsp.gr/iplr

^6^The other variants (V2 and N2) were also used as targets in this experiment, in fewer trials, as a pilot investigation of priming symmetry. Results for these targets are not reported, as these were subsequently fully explored in Experiment 2.

^7^In a morphologically rich language like Greek, in which all words are morphologically structured, there are no pseudosuffixed or non-suffixed words to form pseudo-morphologically or morpho-orthographically related word pairs (similar to the English example corner- CORN) or a proper control condition (similar to the English example dialÎ¿g- DIAL) (see Rastle and Davis, 2008 for a review). Consequently, a distinction between morpho-orthography and orthography does not apply in Greek.
